# Latent class analysis of intrinsic capacity among rural older adults in China and its influencing factors

**DOI:** 10.3389/fpubh.2025.1708977

**Published:** 2026-01-14

**Authors:** Zhihui Wang, Hu Jiang, Yue Peng, Jianxia Luo, Weiping Yan

**Affiliations:** The First People's Hospital of Zunyi, The Third Affiliated Hospital of Zunyi Medical University, Zunyi, China

**Keywords:** advanced age, cross-sectional study, frailty, intrinsic capacity, latent class analysis, rural residence, self-care

## Abstract

**Background:**

The decline of intrinsic capacity (IC) is especially pronounced among rural older adults and significantly impacts their health outcomes. However, the heterogeneity of IC profiles in this population and its associations with factors like self-care ability are not well understood.

**Objective:**

This study aimed to identify latent classes of IC among rural older adults in China and examine their influencing factors.

**Methods:**

Using convenience sampling method, 607 rural older adults in Guizhou Province were recruited between January to June, 2025. Data were collected through questionnaires including a general information survey, self-care ability scale, frailty assessment tool, and intrinsic capacity evaluation instrument. Latent class analysis was performed to identify patterns of intrinsic capacity, followed by multivariate logistic regression to determine influencing factors.

**Results:**

The intrinsic capacity of rural older adults was classified into three latent classes: “Physically Weak and Lacking Vitality Group” (38.71%), “Balanced and Stable Group” (22.41%), and “Cognitive-Motor Impairment Group” (38.88%). Multivariate logistic regression analysis revealed that living alone, low monthly household income, advanced age, frailty status, self-care ability, current work participation, rural residence, and multiple medication use were significant influencing factors for the latent classes of intrinsic capacity (*p* < 0.05).

**Conclusion:**

The deterioration of intrinsic capacity among rural older adult populations exhibits distinct categorical patterns. Healthcare providers should implement tailored and individualized nursing interventions based on these differential influencing factors to enhance the intrinsic capacity levels of rural older adults.

## 1 Introduction

Population aging has become a major global public health and social challenge. According to the United Nations, the number of persons aged 65 years or over worldwide is projected to double by 2050, reaching over 1.5 billion, with the most rapid growth occurring in low- and middle-income countries (1). China, as one of the fastest-aging nations, had 264 million people aged 60 and above according to the Seventh National Population Census in 2020, accounting for 18.7% of its total population (2). In response, the Chinese government has elevated healthy aging to a national strategy, with a focus on building an integrated older adults health service system covering health promotion, disease prevention, treatment, rehabilitation, and long-term care (3).

The World Health Organization (WHO) has proposed a functional ability framework for healthy aging, shifting the focus from disease-centered models to the maintenance of intrinsic capacity (IC) (4). IC is defined as the composite of all an individual’s physical and mental capacities, encompassing the domains of cognition, psychology, sensory function, locomotion, and vitality (5). Evidence suggests that decline in IC is a strong predictor of adverse health outcomes, including disability, dependency, and mortality (6). For instance, a longitudinal study have shown that lower IC levels are associated with increased risk of falls and higher three-year mortality among older adults (7).

Despite growing research on IC, most studies have been conducted in urban or clinical settings, leaving rural populations underrepresented. Rural older adults often face distinct challenges such as limited healthcare access, lower socioeconomic status, and higher prevalence of chronic conditions, which may differentially influence IC trajectories (8). In China, rural older adults have been reported to have a 1.36 times higher prevalence of IC decline compared to their urban counterparts (9). This disparity underscores the need for targeted research in rural contexts.

Guizhou Province, a mountainous region in southwestern China with a relatively underdeveloped rural economy, provides a pertinent setting for such research. In 2020, 15.38% of its population was aged 60 and above, with Zunyi City in Guizhou reporting an aging rate of 17.05% (10). The rural older adults in this region are likely to exhibit distinct IC profiles due to geographical, economic, and healthcare resource constraints.

Existing studies on IC have often examined its domains separately, lacking a person-centered approach that captures the heterogeneity in how these domains co-occur. Latent class analysis (LCA) offers a methodological advantage by identifying unobserved subgroups with similar patterns of IC characteristics (11). While one study in Beijing community-dwelling older adults identified three latent classes of IC (12), no such analysis has been conducted among rural Chinese older adults.

Furthermore, factors such as frailty and self-care ability are closely related to IC but are often studied in isolation. Frailty reflects decreased physiological reserve and heightened vulnerability to stressors (13), while self-care ability pertains to the capacity to perform activities essential for maintaining health and well-being (14). Both may interact with IC in shaping functional outcomes, yet their associations with IC patterns in rural older adults remain unclear.

Therefore, this study aimed to identify latent classes of intrinsic capacity among rural older adults in Guizhou, China, using LCA. Secondly, we aimed to examine the influencing factors of these latent classes, including sociodemographic characteristics, health status, frailty, and self-care ability. The findings will provide evidence for developing tailored interventions to maintain or improve IC in vulnerable rural older adult populations, thereby supporting the goals of healthy aging in China.

## 2 Methods

### 2.1 Study design

A cross-sectional study was conducted through an online survey through convenient sampling method. The study was designed and reported in alignment with the Strengthening the Reporting of Observational Studies in Epidemiology (STROBE) guidelines (15).

### 2.2 Sample size estimation

The sample size for this study was determined with consideration for the requirements of Latent Class Analysis. Methodological guidelines and simulation studies suggest that LCA requires an adequate sample size to ensure accurate parameter estimation and stable class solutions. A widely accepted heuristic is to have at least 50 to 100 participants per anticipated latent class (16). Based on prior research in similar populations (12), we anticipated identifying approximately 3 to 5 distinct latent classes of intrinsic capacity. Therefore, the minimum required sample size was estimated to be between 150 (3 classes × 50) and 500 (5 classes × 100) participants. To ensure robust model fit, higher classification accuracy, and account for potential invalid responses, we aimed to recruit a sample size exceeding the upper threshold of this range.

### 2.3 Participants

This cross-sectional study was conducted from January to June 2025 in Zunyi City, Guizhou Province. To ensure socioeconomic and geographic diversity, participants were recruited via consecutive enrollment from older adults (aged ≥60 years) attending routine medical visits or participating in basic public health services at multiple township health centers and village clinics. These facilities were located in both central urban districts and outlying rural counties of Zunyi. The recruitment and screening procedure comprised three steps: (1) Initial contact and age screening for all eligible visitors at each site; (2) Face-to-face eligibility assessment by uniformly trained researchers based on predefined criteria; (3) Obtaining written informed consent after a detailed study explanation. Inclusion criteria: ① Age ≥60 years; ② Possessing basic communication skills and ability to cooperate; ③ Willing and cognitively capable to provide informed consent. Exclusion criteria: ① Severe psychiatric disorders; ② Uncontrolled major metabolic diseases or organ failure; ③ Severe physical disability precluding assessment completion.

### 2.4 Measurements

#### 2.4.1 Demographics

Based on the study objectives, the researchers designed the demographic section to include basic personal information of older people, such as gender, age, family residence location, ethnicity, marital status, source of income, and Health insurance etc.

#### 2.4.2 Intrinsic capacity assessment tools

##### 2.4.2.1 Motor function

We used the Short Physical Performance Battery (SPPB) to measure motor function (17). It includes: (1) a 4-meter gait speed test (participants walked at their usual pace over an 8-meter course with acceleration/deceleration zones; time was converted to a 0–4 point score); (2) a five-repetition sit-to-stand test for lower limb strength (time converted to a 0–4 point score); and (3) a balance test (maintaining side-by-side, semi-tandem, and tandem stances, scored 0–4). A total SPPB score ≤ 8 indicated declined motor function.

##### 2.4.2.2 Sensory function

Investigators reviewed the community health records management system to obtain vision and hearing test results for older adults from the previous year. In cases where participants self-reported a decline in vision and/or hearing that impacted their daily activities, this was documented as declined sensory function.

##### 2.4.2.3 Cognition

Cognitive function was assessed using the Mini-Mental State Examination (MMSE) (18), which evaluates four domains: orientation (10 points), memory and recall (6 points), attention and calculation (5 points), and language abilities (9 points). Each correct response was awarded 1 point, yielding a maximum possible score of 30 points. The cutoff scores for cognitive impairment were education-dependent: ≤17 for illiterate participants, ≤20 for those with primary education, and ≤24 for individuals with junior high school education or higher. Participants scoring below these education-specific thresholds were classified as having cognitive impairment.

##### 2.4.2.4 Vitality

The Short-Form Mini-Nutritional Assessment (MNA-SF) (19) was used for evaluation. It consists of six domains: dietary intake and weight changes, mobility, acute disease or stress, neuropsychological status, and body mass index (BMI). Each domain is scored from 0 ~ 3 points, with a maximum total score of 14 points. A total score of ≤11 points was classified as declined vitality.

##### 2.4.2.5 Psychological domain

The 15-item Geriatric Depression Scale (GDS-15) (20) was used to assess depressive symptoms over the past week. The scale consists of 15 items requiring “yes” or “no” responses, with each depressive response scored as 1 point. Total scores range from 0 ~ 15, with higher scores indicating more severe depressive symptoms. A score≥8 points was defined as declined psychological function.

#### 2.4.3 Exercise of self-care agency scale (ESCA)

ESCA (21) was jointly developed by Fleischer et al. In 2000, scholars from Taiwan, China, translated the scale into Chinese (22). The Chinese version demonstrated excellent psychometric properties: content validity index of 1.0, internal consistency reliability of 0.86 ~ 0.92, test–retest re-liability of 0.91, and Cronbach’s alpha coefficients ranging from 0.77 ~ 0.80. The scale comprises 43 items across four dimensions: health knowledge level (17 items), self-concept (8 items), self-care responsibility (6 items), self-care skills (12 items). Items are rated on a 5-point Likert scale (0 ~ 4 points per item). Notably, 11 items are reverse-scored, while the remaining items are positively scored. The total score ranges from 0 ~ 172 points, the higher score, the better self-care ability.

#### 2.4.4 Fatigue, resistance, ambulation, illnesses and loss of weight (FRAIL)

The scale consists of 5 items, each scored as 0 or 1, with a total score ranging from 0 to 5. Higher scores indicate greater frailty severity. Specific items include: Fatigue: Whether the individual frequently felt tired or weak in the past 4 weeks Resistance: Whether the individual could independently rise from a chair. Mobility: Whether the individual could walk 100 meters without assistance. Chronic diseases: Whether the individual had 5 or more chronic conditions. Weight loss: Whether the individual experienced unintentional weight loss of ≥5% in the past year. Scoring interpretation: 0 points = Robust (no frailty), 1 ~ 2 points = Pre-frail, 3 ~ 5 points = Frail. The scale demonstrated good content validity (0.878) and acceptable internal consistency (Cronbach’s *α* = 0.75). Test–retest reliability was 0.77, indicating satisfactory stability over time. The scale demonstrates good reliability and validity (23).

### 2.5 Data collection

Data were collected on-site by a uniformly trained medical team through two primary methods: (1) Standardized Physical Examinations: Team members measured and recorded physiological indicators, according to strict operational protocols. (2) Structured Questionnaire Assessment: A comprehensive assessment was administered via face-to-face interviews using electronic devices. To ensure accuracy and accommodate potential literacy or comprehension challenges among rural older adults, researchers read each question aloud and entered responses directly based on participants’ answers. The questionnaire covered domains of intrinsic capacity, frailty, and self-care ability.

### 2.6 Statistical analyses

Data analysis was performed using Mplus 8.3 and SPSS 26.0 software. Latent class analysis (LCA) was employed to examine the intrinsic capacity of rural older adults. Model fit was evaluated based on the Akaike Information Criterion (AIC), Bayesian Information Criterion (BIC), and sample-adjusted Bayesian Information Criterion (aBIC), with lower values indicating better fit. The classification accuracy of the model was assessed using Entropy. The Lo–Mendell–Rubin likelihood ratio test (LMR) and the Bootstrapped Likelihood Ratio Test (BLRT) were applied, where a significant *p*-value (<0.05) suggested that the model with an additional class provided a better fit. Starting with a single-class model, the number of latent classes was incrementally increased, and the optimal model was selected by comprehensively considering all fit indices. For data description, normally distributed continuous variables were presented as mean±standard deviation (SD), while non-normally distributed continuous variables were described using M (P25, P75). Categorical variables were summarized as frequencies and percentages. Group comparisons were conducted using the χ^2^/Fisher’s exact test (for nominal variables), the Kruskal-Wallis *H* test (for ordinal variables), and analysis of variance (ANOVA). Multivariable logistic regression was performed to explore the influencing factors of latent classes of intrinsic capacity among rural older adults. A two-sided *p* < 0.05 was considered statistically significant.

### 2.7 Ethical considerations

The Ethics Committee of the Third Affiliated Hospital of Zunyi Medical University (The First People’s Hospital of Zunyi) (2022-1-124). Before accessing the survey, participants were presented with information about the study’s purpose and potential benefits on the web-based questionnaire platform. Rural older adults could then choose to either agree or decline participation. The study was conducted anonymously, ensuring that participants’ information would remain confidential and be used exclusively for scientific research purposes. Participants had the right to withdraw from the survey at any time without facing any consequences.

## 3 Results

A total of 620 questionnaires were distributed in this study. After data cleaning, 13 (2.1%), invalid questionnaires were excluded, and 607 (97.9%) valid questionnaires were ultimately obtained for subsequent statistical analysis.

### 3.1 Latent class analysis on intrinsic capacity among rural older adults

A latent class analysis was conducted on the intrinsic capacity of rural older adults participating in the baseline survey, with five latent class models fitted (Table 1). While the 2-class model showed the most favorable entropy, the Akaike Information Criterion (AIC), Bayesian Information Criterion (BIC), and adjusted BIC values continued to decrease with increasing class numbers, leading to the exclusion of the 2-class solution. After comprehensive evaluation, the 3-class model was selected as optimal. The average probabilities of participants being assigned to their respective classes were 96, 88.6, and 94.6%, indicating high classification reliability.

**Table 1:** Comparison of fit indices between models.

Class	AIC	BIC	aBIC	LMR	BLRT	Entropy	Category probability
1	3445.588	3467.631	3451.757	-	-		1
2	3285.098	3333.591	3298.669	<0.001	<0.001	0.656	0.77265/0.22735
3	3261.721	3336.666	3282.695	<0.001	<0.001	0.612	0.38715/0.22405/0.38880
4	3265.558	3366.954	3293.934	0.329	0.667	0.661	0.30972/0.24053/0.31631/0.13344

**Figure 1:**
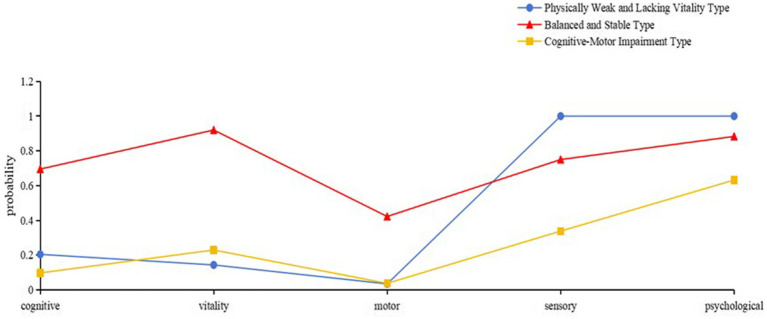
Characteristics distribution of the three latent classes of intrinsic capacity among rural older adults.

The 3-class model categorized rural older adults’ intrinsic capacity into three distinct profiles: C1 (38.71%, *n* = 235): Characterized by low conditional probabilities in vitality and motor function, hence labeled as “Physically Weak and Lacking Vitality Group.” C2 (22.41%, *n* = 136): Demonstrated high conditional probabilities (>0.700) across psychological, sensory, vitality, and cognitive domains, designated as “Balanced and Stable Group.” C3 (38.88%, *n* = 236): Showed reduced probabilities in motor and cognitive functions, termed “Cognitive-Motor Impairment Group.” The results were presented in Figure 1.

### 3.2 Characteristics of intrinsic capacity in rural older adults

Among rural older adults, 94.73% (*n* = 575) exhibited declined intrinsic capacity. Domain-specific prevalence rates were: psychological (20.7%, *n* = 126), sensory (38.3%, *n* = 233), vitality (61.6%, *n* = 374), motor (86.4%, *n* = 525), and cognitive (72.1%, *n* = 438) declines. Significant differences (*p* < 0.05) were observed across latent classes in age, education level, marital status, living arrangements, ESCA scores, and FRAIL scale results. The results were presented in Table 2.

**Table 2:** Comparison of characteristics across different groups.

Variables	Physically weak and lacking vitality group (*n* = 235)	Balanced and stable group (*n* = 136)	Cognitive-motor impairment group (*n* = 236)	F/χ^2^ *H*/*Z*	*p*
Gender *n* (%)				2.22	0.329
Male	135 (57.45)	71 (52.21)	120 (50.85)		
Female	100 (42.55)	65 (47.79)	116 (49.15)		
Ethnicity *n* (%)				68.03	**<0.001**
Han Chinese	184 (78.30)	50 (36.76)	124 (52.54)		
Ethnic minorities	51 (21.70)	86 (63.24)	112 (47.46)		
Current Level of Education *n* (%)				-	**<0.001**
High school and above	1 (0.43)	2 (1.47)	3 (1.27)		
Never attended school	76 (32.34)	49 (36.03)	173 (73.31)		
Primary or junior high school	158 (67.23)	85 (62.50)	60 (25.42)		
Marital status *n* (%)				126.85	<0.001
Married	215 (91.49)	117 (86.03)	114 (48.31)		
Single	20 (8.51)	19 (13.97)	122 (51.69)		
Residential group *n* (%)				7.87	**0.020**
Living alone	39 (16.60)	38 (27.94)	59 (25.00)		
Living with family	196 (83.40)	98 (72.06)	177 (75.00)		
Family residence *n* (%)				36.48	**<0.001**
Urban	7 (2.98)	30 (22.06)	21 (8.90)		
Rural	228 (97.02)	106 (77.94)	215 (91.10)		
Mean monthly family income (RMB) *n* (%)				53.46	**<0.001**
<1,000	17 (7.23)	16 (11.76)	29 (12.29)		
1,000–3,000	98 (41.70)	47 (34.56)	87 (36.86)		
3,001–5,000	116 (49.36)	41 (30.15)	95 (40.25)		
>5,001	4 (1.70)	32 (23.53)	25 (10.59)		
Income source *n* (%)				88.49	**<0.001**
Other	21 (8.94)	11 (8.09)	4 (1.69)		
Retirement pension	5 (2.13)	5 (3.68)	5 (2.12)		
Agricultural income	122 (51.91)	67 (49.26)	57 (24.15)		
Old-age pension	1 (0.43)	9 (6.62)	5 (2.12)		
Support from children or others	86 (36.60)	44 (32.35)	165 (69.92)		
Medical insurance *n* (%)				-	0.214
Resident insurance	234 (99.57)	133 (97.79)	231 (97.88)		
Employee insurance	1 (0.43)	3 (2.21)	5 (2.12)		
Smoking *n* (%)				2.19	0.335
No	163 (69.36)	85 (62.50)	152 (64.41)		
Yes	72 (30.64)	51 (37.50)	84 (35.59)		
Alcohol consumption *n* (%)				12.48	**0.002**
No	194 (82.55)	97 (71.32)	163 (69.07)		
Yes	41 (17.45)	39 (28.68)	73 (30.93)		
Regular exercise *n* (%)				25.55	**<0.001**
No	225 (95.74)	111 (81.62)	222 (94.07)		
Yes	10 (4.26)	25 (18.38)	14 (5.93)		
Currently engaged in labor *n* (%)				58.38	**<0.001**
No	107 (45.53)	43 (31.62)	166 (70.34)		
Yes	128 (54.47)	93 (68.38)	70 (29.66)		
Denture status *n* (%)				-	**<0.001**
Partial denture	24 (10.21)	21 (15.44)	84 (35.59)		
Full denture	2 (0.85)	1 (0.74)	5 (2.12)		
No denture	209 (88.94)	114 (83.82)	147 (62.29)		
Hypertension *n* (%)				33.96	**<0.001**
No	193 (82.13)	77 (56.62)	146 (61.86)		
Yes	42 (17.87)	59 (43.38)	90 (38.14)		
Diabetes *n* (%)				19.26	**<0.001**
No	220 (93.62)	107 (78.68)	196 (83.05)		
Yes	15 (6.38)	29 (21.32)	40 (16.95)		
Multiple medication use *n* (%)				31.30	**<0.001**
No	228 (97.02)	107 (78.68)	204 (86.44)		
Yes	7 (2.98)	29 (21.32)	32 (13.56)		
Age, M (P_25_, P_75_)	68.00 (65.00, 72.00)	66.00 (62.00, 70.00)	75.00 (70.00, 80.00)	138.89	**<0.001**
BMI, M (P_25_, P_75_)	20.20 (18.99, 21.64)	22.80 (21.07, 24.60)	20.00 (18.09, 21.75)	98.56	**<0.001**
Dominant handgrip strength, M (P_25_, P_75_)	19.00 (14.50, 25.00)	25.00 (24.00, 30.00)	15.00 (10.00, 20.00)	140.39	**<0.001**
Charlson comorbidity index, M (P_25_, P_75_)	0.00 (0.00, 1.00)	1.00 (0.00, 1.00)	1.00 (0.00, 1.00)	59.43	**<0.001**
Frail, M (P_25_, P_75_)	0.00 (0.00, 1.00)	0.00 (0.00, 1.00)	2.00 (1.00, 3.00)	172.69	**<0.001**

The bold values indicate statistically significant differences.

### 3.3 Comparative analysis of ESCA between different groups

The results showed that the balanced and stable group scored significantly higher than the other two groups across all four dimensions—health knowledge, self-concept, self-care responsibility, and skills, whereas the physically weak and lacking vitality group and the cognitive-motor impairment group performed similarly in most dimensions. The results were presented in Table 3.

**Table 3:** Comparative analysis of ESCA between different groups.

Variables	Physically weak and lacking vitality group (*n* = 235)	Balanced and stable group (*n* = 136)	Cognitive-motor impairment group (*n* = 236)	*H*	*Post-hoc*
Health knowledge level	37.00 (34.00, 39.50)	38.00 (34.00, 41.00)	35.00 (32.00, 39.00)	20.61	**2>1>3**
Self-concept	16.00 (15.00, 18.00)	18.00 (16.00, 20.00)	16.00 (14.00, 18.00)	39.58	**2>1 ≈ 3**
Self-care responsibility	12.00 (11.00, 13.00)	13.00 (9.00, 15.00)	12.00 (10.00, 13.00)	6.11	**2>1 ≈ 3**
Self-care skills	24.00 (23.00, 26.00)	27.00 (24.00, 32.00)	24.00 (22.00, 27.25)	30.08	**2>1 ≈ 3**
ESCA	89.00 (86.00, 94.00)	96.00 (88.00, 102.00)	87.00 (82.75, 95.00)	46.82	**2>1>3**

In the aforementioned chart, Group 2 (Balanced and stable group) scored the highest across all ESCA dimensions and showed statistically significant differences compared to Group 1 and Group 3 (all H-values indicate significant inter-group differences). Except for the Health Knowledge Level and Esca total score, there were no significant differences in scores between Group 1 and Group 3 in the dimensions of Self-Concept, Self-Responsibility, and Self-Care Skills (Group 1 ≈ Group 3). In the dimensions of Health Knowledge Level and Esca total score, the ranking of scores was Group 2 > Group 1 > Group 3, with significant differences observed among all three groups.

### 3.4 Multivariate analysis of different latent classes

Using the latent classes of intrinsic capacity as the dependent variable, we performed multivariate logistic regression analysis incorporating all indicators with *p* < 0.05 in univariate analysis, along with FRAIL and ESCA total scores as independent variables. The results identified age, ethnicity, living arrangement, residential location, monthly household income, current work participation, Multiple Medication Use (≥2 medications), FRAIL score, and ESCA score as statistically significant influencing factors (*p* < 0.05) for intrinsic capacity classification. The results were presented in Table 4.

**Table 4:** Logistic regression analysis of factors influencing intrinsic capacity latent classes in rural older adults.

Variables		Physically weak and lacking vitality group VS Balanced and stable group					Cognitive-motor impairment group VS Balanced and stable group				
		*β*	SD	Wald	OR (95% CI)	*p*	*β*	SD	Wald	OR (95% CI)	*p*
Age		0.037	0.019	3.695	1.037 (0.999–1.077)	0.055	0.044	0.021	4.429	1.045 (1.003–1.089)	0.035
FRAIL		0.052	0.174	0.090	1.054 (0.75–1.481)	0.764	0.645	0.162	15.790	1.906 (1.387–2.619)	<0.001
ESCA		−0.039	0.015	7.111	0.961 (0.934–0.99)	0.008	−0.064	0.016	16.255	0.938 (0.910–0.968)	<0.001
Residential group	Living alone	−0.932	0.326	8.169	0.394 (0.208–0.746)	0.004	0.039	0.349	0.012	1.039 (0.524–2.061)	0.912
	Living with family	Reference					Reference				
Family residence	Rural	2.412	0.588	16.800	11.152 (3.52–35.333)	<0.001	1.074	0.505	4.523	2.927 (1.088–7.875)	0.033
	Urban	Reference					Reference				
Mean monthly family income (CNY)	<1,000	1.946	0.776	6.296	7.001 (1.531–32.01)	0.012	0.694	0.643	1.167	2.002 (0.568–7.054)	0.280
	1,000–3,000	1.778	0.667	7.100	5.92 (1.6–21.901)	0.008	0.557	0.551	1.023	1.745 (0.593–5.137)	0.312
	3,001–5,000	1.757	0.659	7.109	5.795 (1.593–21.086)	0.008	0.734	0.549	1.786	2.084 (0.71–6.115)	0.181
	>5,001	Reference					Reference				
Currently engaged in labor		−0.847	0.338	6.270	0.429 (0.221–0.832)	0.012	−1.013	0.355	8.155	0.363 (0.181–0.728)	0.004
Multiple medication use (≥2)		−1.726	0.586	8.683	0.178 (0.057–0.561)	0.003	−1.194	0.500	5.692	0.303 (0.114–0.808)	0.017

## 4 Discussion

The primary objective of this study was to analyze the latent classes and influencing factors of intrinsic capacity among rural older adult populations. This study identified three distinct latent classes of intrinsic capacity among rural older adults in China: “Physically Weak and Lacking Vitality Group” (38.7%), “Balanced and Stable Group” (22.4%), and “Cognitive-Motor Impairment Group” (38.9%). This classification underscores the heterogeneity of IC decline in this population. Factors significantly associated with class membership included socioeconomic factors (living alone, lower income, rural residence), health status (frailty, multiple medication use), and functional factors (self-care ability, current work participation).

The study revealed the latent class analysis was conducted on the intrinsic capacity of rural older adults participating in the baseline survey, with five latent class models fitted. While the 2-class model showed the most favorable entropy, the AIC, BIC, and adjusted BIC values continued to decrease with increasing class numbers, leading to the exclusion of the 2-class solution. After comprehensive evaluation, the 3-class model was selected as optimal. Consistent with previous studies, latent class analysis in this research also categorized older adults’ intrinsic capacity into three groups, with all groups demonstrating the critical differentiating roles of sensory function and cognitive function. While prior studies primarily focused on sensory function-dominated classifications, the current study highlights the dimension of physical function. Moreover, this study identified a distinct “Balanced and Stable Group,” which was not separately distinguished in previous research (12). In this research, although the “Balanced and Stable Group” of rural older adults exhibited a high overall functional level, most had already experienced declines in vitality and mobility. The study found that declines in vitality and mobility may predict the likelihood of deterioration in other domains of intrinsic capacity (24). Healthcare providers can train this group in accessing online health resources and maintaining social ties, fostering active engagement and self-driven capacity preservation. With aging, older adults commonly develop sensory and physical impairments, increasing their fall risk and activity avoidance, which accelerates physical decline (25). For the “Physically Weak and Lacking Vitality Group” of rural older adults, healthcare professionals can design chair-based exercises (26) and resistance training to enhance their muscle strength and balance. Additionally, cognitive decline in rural older adults is often accompanied by depression (27). For individuals in the Cognitive-Motor Impairment Group, regular psychological monitoring should be implemented, combined with appropriately timed physical training and digital cognitive interventions. These measures aim to enhance cognitive function and bolster resilience in coping with challenges (28). Compared to existing research on the intrinsic capabilities of rural older adults, the categorization in this study both validates certain common findings and provides new insights (29).

The intrinsic capacity of rural older adults varies across different categories due to the influence of demographic characteristics. The study revealed that rural older adults living alone with a household monthly income below 5,000 CNY are more likely to belong to the “Physically Weak and Lacking Vitality Group” group. Compared to those living with spouses or children, who exhibit higher intrinsic capacity likely due to better material, emotional, and caregiving support. Older adults who are divorced, separated, or widowed often face inadequate social support and active lifestyles, hindering access to healthcare and worsening health outcomes. Yoon (30) highlighted that solitary living exacerbates depression due to limited familial support. Aligning with WHO’s Integrated Care for Older People guidelines (31), cohabitation may promote healthy aging through holistic care involvement. Lower income significantly correlates with diminished intrinsic capacity (vitality, mobility) via malnutrition, limited healthcare access, and chronic stress (32, 33), while financial constraints also impede chronic disease management, accelerating functional decline (12). To address these issues, hospitals and policymakers should strengthen rural older adult support systems (pensions, health insurance, care subsidies) and adopt “smart aging” initiatives (digital “Internet + older adult care” platforms) to enhance service efficiency. Multidimensional interventions targeting economic and healthcare barriers can improve intrinsic capacity and quality of life.

The results of our study indicated a correlation among rural older adults with advanced age and frailty diagnosis with intrinsic capacity. The prevalence of visual and hearing impairments among older adults increases exponentially with age (34). Although age is an unmodifiable risk factor, healthcare providers can mitigate its effects through regular audiological assessments, stereoscopic vision tests, and motor function screenings. Sensory compensation techniques and assistive devices can help address sensory and mobility deficits, thereby enhancing intrinsic capacity in rural older adult populations. Research indicates a bidirectional relationship between frailty and cognitive/motor functions in older adults (2). On one hand, frailty, particularly social and cognitive frailty accelerates cognitive decline and increases the risk of motor cognitive risk syndrome and dementia (9). On the other hand, reduced cognitive function and mobility impairments (gait abnormalities due to sarcopenia) further exacerbate frailty, creating a vicious cycle (35). To address this, comprehensive interventions are recommended: Social Engagement: Community activities and family support to reduce loneliness and strengthen social connections; Physical Training: Resistance exercises, balance training (Tai Chi), and aerobic activities to improve motor function; Early Screening: Frailty scales and smart monitoring technologies for timely detection and intervention. These strategies aim to slow functional decline and improve quality of life for aging populations.

Our findings indicated a correlation between self-care abilities and intrinsic capacity in older adults. Higher self-care ability helps maintain and enhance all five domains of intrinsic capacity, while a decline in intrinsic capacity, in turn, weakens an older adult’s ability to perform self-care (36). Studies indicate that older adults with greater self-care abilities tend to report better health, stronger social support, and less depression, all supporting maintained cognitive, physical, and mental health. Conversely, older adults with weaker self-care capacity, such as those in institutional care settings, often face a higher risk of intrinsic capacity decline due to increased dependency and insufficient health management (37). To strengthen this relationship, community-based health education can improve older adults’ self-care knowledge and skills. Additionally, integrated medical and older adult care models, such as hospital-managed nursing homes providing professional healthcare support can significantly enhance self-care capacity. Through these combined interventions, the decline in intrinsic capacity can be delayed, promoting healthy aging.

Two major research implications emerge from this study. On the one hand, overall trends show rural older adults maintain relative advantages in certain intrinsic capacity domains. Their sustained high-intensity physical labor and active lifestyles provide continuous conditioning for physiological functions. Daily activities like farming and household chores help preserve muscle strength, joint flexibility, and cardiopulmonary function. In contrast, urban older adults’ greater reliance on modern conveniences results in lower daily activity levels, leading to earlier onset of sarcopenia and joint stiffness (38). Consequently, rural residents generally demonstrate better-preserved intrinsic capacity. On the other hand, multiple medication use elevates intrinsic capacity decline risk, likely due to chronic functional impairment in multimorbid patients. In low- and middle-income countries, over 50% of older adults require multiple concurrent medications for comorbid conditions (39). As emphasized in WHO’s World Report on Aging and Health (4), functional ability must remain a priority even when managing chronic diseases. It should also be noted that an individual’s health status is dynamic. When assessing the health needs of older adults, in addition to considering the specific diseases they may be experiencing, it is essential to account for how the interactions between these conditions affect functional ability.

### 4.1 Limitations

Although this study systematically analyzed the latent classes of intrinsic capacity among rural older adults, several limitations should be acknowledged. First, the study focused solely on rural older adults in specific regions, and its findings may not be generalizable to other areas of China, warranting further validation of their broader applicability. Additionally, during data collection, assessments of certain sensory and cognitive functions might have been influenced by the relatively low education levels and limited health literacy among rural older adults, potentially introducing measurement bias. Furthermore, the analysis of socioeconomic factors such as income revealed a nonlinear relationship with intrinsic capacity. The limited sample size in the low-income group may have amplified the estimation error of associated risks, while the notably higher proportion of high-income individuals in the “balanced and stable group” suggests the potential protective role of economic resources for functional health. These findings should be interpreted with caution due to possible measurement constraints and sample distribution characteristics. These limitations highlight the need for future research to enhance sample diversity, develop culturally adapted assessment tools, refine socioeconomic measurements (including income sources and stability), and validate intervention effectiveness.

## 5 Conclusion

The study identified three distinct patterns of intrinsic capacity decline among rural Chinese older adults through latent class analysis. The study found that rural older adults with lower income or living alone face higher risks of vitality loss and mobility decline, while older age and pre-existing frailty correlate with both cognitive and motor deterioration. We recommend multidisciplinary interventions, including IC assessment, rehabilitation, psychological support, and tailored nursing, to preserve function and improve quality of life. Future research should focus on tracking longitudinal trajectories of IC changes in rural older adults, with particular emphasis on identifying modifiable risk factors for functional decline to inform the development of precise, differentiated rural older adult healthcare strategies.

## Data Availability

The raw data supporting the conclusions of this article will be made available by the authors, without undue reservation.

## References

[1] Beard JR Si Y Liu Z Chenoweth L Hanewald K. Intrinsic capacity: validation of a new WHO concept for healthy aging in a longitudinal Chinese study. J Gerontol A Biol Sci Med Sci. (2022) 77:94–100. doi: 10.1093/gerona/glab226, 34343305

[2] Zhou Y Ma L. Intrinsic capacity in older adults: recent advances. Aging Dis. (2022) 13:353–9. doi: 10.14336/AD.2021.0818, 35371613 PMC8947834

[3] Liu Y Du Q Jiang Y. Detection rate of decreased intrinsic capacity of older adults: a systematic review and meta-analysis. Aging Clin Exp Res. (2023) 35:2009–17. doi: 10.1007/s40520-023-02515-7, 37543528

[4] World Health Organization. World report on ageing and health. Geneva: World Health Organization (2015).

[5] Cesari M Araujo de Carvalho I Amuthavalli Thiyagarajan J Cooper C Martin FC Reginster J-Y . Evidence for the domains supporting the construct of intrinsic capacity. J Gerontol A Biol Sci Med Sci. (2018) 73:1653–60. doi: 10.1093/gerona/gly011, 29408961

[6] González-Bautista E de Souto BP Andrieu S Rolland Y Vellas B. Screening for intrinsic capacity impairments as markers of increased risk of frailty and disability in the context of integrated care for older people: secondary analysis of MAPT. Maturitas. (2021) 150:1–6. doi: 10.1016/j.maturitas.2021.05.011, 34274071

[7] Charles A Buckinx F Locquet M Reginster J-Y Petermans J Gruslin B . Prediction of adverse outcomes in nursing home residents according to intrinsic capacity proposed by the World Health Organization. J Gerontol A Biol Sci Med Sci. (2020) 75:1594–9. doi: 10.1093/gerona/glz218, 31562812

[8] Yu S Wang J Xia Y Tang Q. The status quo and influencing factors of intrinsic capacity among community-dwelling older adults from the perspective of ecological systems theory: a cross-sectional study. BMC Geriatr. (2024) 24:934. doi: 10.1186/s12877-024-05499-9, 39533175 PMC11555801

[9] Liu S Kang L Liu X Zhao S Wang X Li J . Trajectory and correlation of intrinsic capacity and frailty in a Beijing elderly community. Front Med. (2021) 8:751586. doi: 10.3389/fmed.2021.751586, 34957141 PMC8695757

[10] Lulin H. Impact of population aging trends on economic development in Guizhou Province and research-based countermeasures. Chin Market. (2025) 5:21–4.

[11] Nylund-Gibson K Choi AY. Ten frequently asked questions about latent class analysis. Transl Issues Psychol Sci. (2018) 4:440–61. doi: 10.1037/tps0000176

[12] Wei X Chen Y Qin J Yang Y Yang T Yan F . Factors associated with the intrinsic capacity in older adults: a scoping review. J Clin Nurs. (2024) 33:1739–50. doi: 10.1111/jocn.17017 38345142

[13] Clegg A Young J Iliffe S Rikkert MO Rockwood K. Frailty in elderly people. Lancet. (2013) 381:752–62. doi: 10.1016/S0140-6736(12)62167-9, 23395245 PMC4098658

[14] Vermeiren S Vella-Azzopardi R Beckwee D Habbig A-K Scafoglieri A Jansen B . Frailty and the prediction of negative health outcomes: a meta-analysis. J Am Med Dir Assoc. (2016) 17:1163.e1–1163.e17. doi: 10.1016/j.jamda.2016.09.010, 27886869

[15] Von Elm E Altman DG Egger M Pocock SJ Gøtzsche PC Vandenbroucke JP. The strengthening the reporting of observational studies in epidemiology (STROBE) statement: guidelines for reporting observational studies. Int J Surg. (2014) 12:1495–9. doi: 10.1016/j.ijsu.2014.07.013, 25046131

[16] Dziak JJ Lanza ST Tan X. Effect size, statistical power, and sample size requirements for the bootstrap likelihood ratio test in latent class analysis. Struct Equ Model Multidiscip J. (2014) 21:534–52. doi: 10.1080/10705511.2014.919819, 25328371 PMC4196274

[17] Guralnik JM Simonsick EM Ferrucci L Glynn RJ Berkman LF Blazer DG . A short physical performance battery assessing lower extremity function: association with self-reported disability and prediction of mortality and nursing home admission. J Gerontol. (1994) 49:M85–94. doi: 10.1093/geronj/49.2.M85, 8126356

[18] Folstein MF Folstein SE McHugh PR. “Mini-mental state”: a practical method for grading the cognitive state of patients for the clinician. J Psychiatr Res. (1975) 12:189–98. doi: 10.1016/0022-3956(75)90026-6, 1202204

[19] Rubenstein LZ Harker JO Salvà A Guigoz Y Vellas B. Screening for undernutrition in geriatric practice: developing the short-form mini-nutritional assessment (MNA-SF). J Gerontol A Biol Sci Med Sci. (2001) 56:M366–72. doi: 10.1093/gerona/56.6.M366, 11382797

[20] Yesavage JA Brink TL Rose TL Lum O Huang V Adey M . Development and validation of a geriatric depression screening scale: a preliminary report. J Psychiatr Res. (1982) 17:37–49. doi: 10.1016/0022-3956(82)90033-4, 7183759

[21] Kearney BY Fleischer BJ. Development of an instrument to measure exercise of self-care agency. Res Nurs Health. (1979) 2:25–34. doi: 10.1002/nur.4770020105, 254279

[22] Wang H-H Laffrey SC. Preliminary development and testing of instruments to measure self-care agency and social support of women in Taiwan. Kaohsiung J Med Sci. (2000) 16:459–67. 11271731

[23] Ng YX Cheng LJ Quek YY Yu R Wu XV. The measurement properties and feasibility of FRAIL scale in older adults: a systematic review and meta-analysis. Ageing Res Rev. (2024) 95:102243. doi: 10.1016/j.arr.2024.102243, 38395198

[24] YuR LaiD LeungG WooJ. Trajectories of intrinsic capacity: determinants and associations with disability. J Nutr Health Aging. (2023) 27:174–81. doi: 10.1007/s12603-023-1881-5, 36973922

[25] Campos J Ramkhalawansingh R Pichora-Fuller MK. Hearing, self-motion perception, mobility, and aging. Hear Res. (2018) 369:42–55. doi: 10.1016/j.heares.2018.03.025, 29661612

[26] Klempel N Blackburn NE McMullan IL Wilson JJ Smith L Cunningham C . The effect of chair-based exercise on physical function in older adults: a systematic review and meta-analysis. Int J Environ Res Public Health. (2021) 18:1902. doi: 10.3390/ijerph18041902, 33669357 PMC7920319

[27] Utne I Cooper BA Ritchie C Wong M Dunn LB Loyland B . Co-occurrence of decrements in physical and cognitive function is common in older oncology patients receiving chemotherapy. Eur J Oncol Nurs. (2020) 48:101823. doi: 10.1016/j.ejon.2020.101823, 32835999 PMC7584738

[28] Li R Geng J Yang R Ge Y Therese H. Effectiveness of computerized cognitive training in delaying cognitive function decline in people with mild cognitive impairment: systematic review and meta-analysis. J Med Internet Res. (2022) 24:e38624. doi: 10.2196/38624 36301590 PMC9650579

[29] Wang X Yang T Li Y Ma C Yang M Qian Q . Intrinsic capacity decline as a predictor of functional disability in the elderly: a systematic review and meta-analysis. Arch Gerontol Geriatr. (2024) 126:105550. doi: 10.1016/j.archger.2024.105550, 38991290

[30] Yoon S Shin C Han C. Depression and cognitive function in mild cognitive impairment: a 1-year follow-up study. J Geriatr Psychiatry Neurol. (2017) 30:280–8. doi: 10.1177/0891988717723741, 28925333

[31] World Health Organization. Integrated care for older people: guidelines on community-level interventions to manage declines in intrinsic capacity. Geneva: World Health Organization (2017). 29608259

[32] Aliberti MJ Bertola L Szlejf C Oliveira D Piovezan RD Cesari M . Validating intrinsic capacity to measure healthy aging in an upper middle-income country: findings from the ELSI-Brazil. Lancet Reg Health Am. (2022) 12:100284. doi: 10.1016/j.lana.2022.100284 36776430 PMC9903598

[33] Sánchez-Sánchez JL Lu W-H Gallardo-Gómez D del Pozo Cruz B de Souto Barreto P Lucia A . Association of intrinsic capacity with functional decline and mortality in older adults: a systematic review and meta-analysis of longitudinal studies. The Lancet Healthy Longevity (2024) 5:e480–e492. doi: 10.1016/s2666-7568(24)00092-8 38945130

[34] Jiang X Chen F Yang X Yang M Zhang X Ma X . Effects of personal and health characteristics on the intrinsic capacity of older adults in the community: a cross-sectional study using the healthy aging framework. BMC Geriatr. (2023) 23:643. doi: 10.1186/s12877-023-04362-7, 37817083 PMC10566030

[35] Merchant RA Chan YH Anbarasan D Vellas B. Association of intrinsic capacity with functional ability, sarcopenia and systemic inflammation in pre-frail older adults. Front Med. (2024) 11:1374197. doi: 10.3389/fmed.2024.1374197, 38510450 PMC10953915

[36] Leung AY Su JJ Lee ES Fung JT Molassiotis A. Intrinsic capacity of older people in the community using WHO integrated care for older people (ICOPE) framework: a cross-sectional study. BMC Geriatr. (2022) 22:304. doi: 10.1186/s12877-022-02980-1, 35395736 PMC8993034

[37] Yoshimura J Tanimura C Matsumoto H Tokushima Y Inoue K Park D . Relationship of physical activity to self-care agency and physical condition among older adults in a rural area. Yonago Acta Med. (2021) 64:18–29. doi: 10.33160/yam.2021.02.004, 33642900 PMC7902176

[38] Moon SW Kim K-J Lee HS Yun YM Kim J-E Chun YJ . Low muscle mass, low muscle function, and sarcopenia in the urban and rural elderly. Scientific Reports (2022) 12:14314. doi: 10.1038/s41598-022-18167-y 35995980 PMC9395512

[39] Arokiasamy P Uttamacharya U Jain K Biritwum RB Yawson AE Wu F . The impact of multimorbidity on adult physical and mental health in low-and middle-income countries: what does the study on global ageing and adult health (SAGE) reveal? BMC Med. (2015) 13:1–16. doi: 10.1186/s12916-015-0402-8, 26239481 PMC4524360

